# Evaluation of Prior Authorization in Medicare Nonemergent Ambulance Transport

**DOI:** 10.1001/jamahealthforum.2022.2093

**Published:** 2022-07-15

**Authors:** Kara Contreary, Andrew Asher, Jared Coopersmith

**Affiliations:** 1 Mathematica, Washington, DC

## Abstract

**Importance:**

Some Medicare-reimbursed services are overused or improperly used, resulting in payments for unnecessary services.

**Objective:**

To determine if prior authorization of services vulnerable to improper use is associated with reduced use and costs without changing patient access or health outcomes.

**Design, Setting, and Participants:**

This study involved repeated cross-sectional evaluation with a state-level matched control group construction and inverse propensity score weighting at the Medicare beneficiary level. Eight states plus the District of Columbia requiring prior authorization were compared with 13 matched comparison group states not subject to prior authorization. Observations on approximately 1.7 million Medicare beneficiaries spanned January 2012 through December 2019. Depending on their state of residence, this included 3 or 4 preintervention years and 4 or 5 postintervention years. Data analysis was performed from September 2020 to July 2021.

**Intervention:**

Ambulance suppliers were directed to request prior authorization for Repetitive, Scheduled, Non-Emergent Ambulance Transport (RSNAT) services; failure to do so resulted in prepayment claim review. The goal of prior authorization is to reduce use of nonemergency ambulance transports that do not meet Medicare coverage criteria.

**Main Outcomes and Measures:**

Primary outcomes included total cost of care, RSNAT use rates and expenditures, unplanned hospital admission, emergency department admission, and emergency ambulance use per beneficiary-year. All measures were constructed from Medicare claims.

**Results:**

Approximately 1.7 million Medicare beneficiaries were observed in the study (mean [SD] age, 71 [15] years; 50% female beneficiaries; 30% Black beneficiaries, 64% White beneficiaries; 20% rural residence; 35% dually eligible for Medicare and Medicaid; 58% with end-stage renal disease; and 44% with severe [stage 3 or 4] pressure ulcers). After controlling for covariates, the results showed that prior authorization was associated with a 2.4% reduction in total annual expenditures for a total of $1530 per beneficiary-year (95% CI, −$1775 to −$1285; *P* < .001); a 61% reduction in the probability of RSNAT use for a total of 4.1 percentage points per beneficiary-year (95% CI, −4.26 to −3.94; *P* < .001); a 77% reduction in RSNAT expenditures for a total of $1136 per beneficiary-year (95% CI, −$1179 to −$1093; *P* < .001); a 1.4% reduction in the probability of emergency department use by 0.99 percentage points per beneficiary-year (95% CI, −1.17 to −0.81; *P* < .001); no change in the probability of emergency ambulance use (0.07 percentage points, 95% CI, −0.15 to 0.29; *P* = .50); a 2.6% reduction in the probability of unplanned hospital admission for a total of 1.53 percentage points per beneficiary-year (95% CI, −1.71 to −1.35; *P* < .001); and a 19% annual increase in the probability of emergency dialysis use for a total of 1.4 percentage points per beneficiary with end-stage renal disease (95% CI, 1.28 to 1.60; *P* < .001).

**Conclusions and Relevance:**

In this difference-in-differences analysis of Medicare beneficiaries, the results suggest that the RSNAT Prior Authorization Model was associated with reduced costs with little or no change in the quality or access indicators examined. Targeted approaches to prior authorization may be an appropriate control measure for Medicare services vulnerable to improper use.

## Key Points

**Question:**

What is the association between a prior authorization requirement for nonemergency ambulance transportation and Medicare costs, patient access to care, and health outcomes for Medicare beneficiaries subject to the Repetitive, Scheduled, Non-Emergent Ambulance Transport Prior Authorization pilot program?

**Findings:**

In this difference-in-differences analysis of 1.7 million Medicare beneficiaries, expenditures for repetitive, scheduled, nonemergency ambulance transport were significantly reduced by 77% and total Medicare expenditures were significantly reduced by 2.4% per beneficiary-year. No clear evidence was found for material changes to patient outcomes.

**Meaning:**

When prior authorization is targeted to services with high improper usage rates, this study suggests that it may be associated with reduced costs without materially changed access to care or patient health.

## Teaser

This cross-sectional study of Medicare beneficiaries assesses whether prior authorization of nonemergency ambulance transports is associated with total cost, patient access, and outcome indicators.

## Introduction

Prior authorization is a strategy used by many private and public health insurers to contain costs and improve the integrity of billing and treatment.^1^ It is used across a wide array of services, including surgeries, hospital inpatient admissions, drugs, home health, and physical therapy. In the Medicare fee-for-service program, the Centers for Medicare & Medicaid Services (CMS) has focused prior authorization on a smaller set of services with documented high levels of improper payments and unnecessary use, which we refer to here as services vulnerable to improper use.

Previous studies found that prior authorization was associated with reduced spending for the targeted medication or service, though in some cases this association waned over time.^1,2,3,4,5,6,7,8,9^ Findings related to the association of prior authorization with total cost of care are mixed, and there is limited evidence on changes in hospitalizations, emergency department visits, time to treatment, patient length of stay, or access to treatment.^1,3,7,10^ By examining total cost of care and a series of patient outcomes and access to care measures, not just spending for the target service, this study is able to assess the potential for targeted prior authorization programs to improve efficiency in the Medicare program without a change in beneficiaries’ health.

This work builds on an analysis the authors prepared for the CMS, which examined the impact of a pilot prior authorization program on nonemergency transport in Medicare on total cost of care and key patient outcomes using a 2-way fixed-effects framework. In this analysis, we use both an event study and a 2-way fixed-effects analysis to better understand whether the implementation of prior authorization is associated with changes in these key outcomes. We consider our findings in the context of the existing literature on prior authorization to address the larger question of whether prior authorization that targets frequently misused services holds potential to reduce total cost of care, while avoiding adverse patient outcomes and compensating cost shifts to other parts of the health care system.

### Program Background

The Medicare Repetitive, Scheduled, Non-Emergent Ambulance Transport Prior Authorization (RSNAT-PA) Model is a pilot program to assess the impact of prior authorization for certain nonemergency ambulance services. The definition of RSNAT is medically necessary transportation by ambulance that occurs 3 times or more during a 10-day period or at least once per week for 3 weeks or longer. Common destinations for Medicare beneficiaries who require RSNAT include dialysis treatment; chemotherapy; and treatment of nonhealing wounds, such as debridement, dressing changes, and hyperbaric oxygen therapy. Medicare coverage rules authorize RSNAT only when beneficiaries are bedbound or otherwise require ambulance transport. Despite the restrictive coverage rules, RSNAT is a highly used service; in 2013, we counted more than 3.9 million RSNAT trips nationwide, an average of more than 10 000 every single day.

Repetitive, scheduled, nonemergent ambulance transport is also prone to improper use. A 2006 US Department of Health and Human Services Office of Inspector General report found that 20% of nonemergency ambulance trips were not properly documented per Medicare guidelines.^11^ A later report found that Medicare paid more than $61 million in just the first 6 months of 2012 for potentially inappropriate ambulance services,^12^ suggesting substantial scope for savings.

The RSNAT-PA Model was intended to reduce ambulance transports that do not meet Medicare coverage criteria. It directs ambulance suppliers located within model states to submit requests for prior authorization along with medical documentation justifying the need for ambulance transport to CMS’s Medicare Administrative Contractor for review. Ambulance suppliers that failed to submit this information had their claims subject to prepayment review.

Implementation of the model began in December 2014 in New Jersey, Pennsylvania, and South Carolina. In January 2016, CMS added 5 more states (Delaware, Maryland, North Carolina, Virginia, and West Virginia) and the District of Columbia. The RSNAT-PA Model ran through December 1, 2020 (with a pause in enforcement during the COVID-19 public health emergency), and on the basis of the findings from the evaluation, CMS announced that it would expand RSNAT-PA nationwide, with rolling start dates in the remaining states and territories.^13^

## Methods

In this difference-in-differences analysis, we used Medicare claims, supplier enrollment, beneficiary eligibility data, and geographic area data from January 2012 through December 2019, which includes 3 years before and 5 years after the model started for the first cohort of states, and 4 years before and 4 years after the model started for the second cohort. Key outcomes included total cost of care (measured as total Medicare expenditures), the probability of RSNAT service use, the number of RSNAT trips, costs for RSNAT services, and several measures of quality and access to care, including emergency department visits, emergency ambulance use, and unplanned hospital admission. Among beneficiaries with end-stage renal disease (ESRD), we also examined scheduled and emergency dialysis use and hospitalization due to ESRD complications. Because we examined CMS administrative data under our contract with CMS, we followed CMS's confidentiality requirements, and institutional review board approval was not required. For this study, we followed the Strengthening the Reporting of Observational Studies in Epidemiology (STROBE) reporting guideline for cross-sectional studies, including details on methods, measurements, and statistical analysis.^14^ Data analysis was performed from September 2020 to July 2021.

### Comparison Group Selection

In keeping with CMS’s state-level implementation of RSNAT-PA, we constructed our comparison group at the state level. Model states were not chosen randomly. Some were selected because of exceptionally high baseline use of ambulance services and high improper payment rates, and the remaining model states were served by the same Medicare administrative contractors as the high-use states. This uniquely high baseline RSNAT use presented a challenge for choosing a valid comparison group. We used the statistical matching technique, optimal full matching, to select a group of states that were as similar as possible to the model states on a range of characteristics.^15,16^ Analyses were performed using R statistical software, version 4.0.2 (R Project for Statistical Computing).^17^ We matched on baseline RSNAT use rates, availability of ambulance suppliers, and rural residence. Matching on rural residence was important because rural areas have more limited ambulance supply and fewer transportation alternatives.

When selecting the comparison group, we matched states to the full set of model states, rather than the 2 separate cohorts. The comparison states selected were Alabama, Florida, Georgia, Indiana, Kentucky, Louisiana, Massachusetts, Montana, Nebraska, Ohio, Tennessee, Texas, and Washington. See eTable 1 in the Supplement for the state-level matching results.

### Study Population and Weighting

We limited our study population to beneficiaries most likely to use RSNAT—those whose service use indicated ESRD and/or severe (stage 3 or 4) pressure ulcers (decubitus ulcers). During the study period, more than 85% of RSNAT users in the study states had 1 or both conditions. Examining RSNAT use among likely users greatly improved our statistical power. We identified beneficiaries with these conditions using Hierarchical Condition Category (HCC) flags (HCC 134 and HCC 136 for ESRD, HCC 157 and HCC 158 for pressure ulcers), which are constructed using claims data.^18^ We included a beneficiary-year if the beneficiary was living in 1 of the study states and enrolled in fee-for-service Medicare in any month in that year. We excluded beneficiaries who moved between study states during the study period, approximately 3% of otherwise qualified beneficiaries.

### Statistical Methods

We used propensity-score weights to balance the demographic and health characteristics of the included beneficiaries living in the comparison states with those living in the model states. These weights were needed for 2 primary reasons. First, state-level matching could not achieve precise balance when examining hundreds of thousands of beneficiaries. Second, the specific set of beneficiaries included in a given analytic year varied because of residential changes, changes in medical coverage, and death. The propensity score weights were designed to balance the comparison beneficiaries with the model-state beneficiaries in each year, and separate weights were calculated for each year given changes in the composition of beneficiaries. The propensity score models included beneficiary age; residence in a rural area; sex; race; indicators for cancer, ESRD, or skin ulcer diagnoses; and an interaction term between age and rural residence. (Race was identified through the Medicare Enrollment Database based on self-reported information originally obtained from the Social Security Administration.) These characteristics were chosen because of their association with many of the outcomes we examined and because they are either fixed demographic characteristics or chronic conditions, both of which are independent of exposure to RSNAT-PA. We conducted our analysis at the beneficiary-year level using SAS, version 9.4 (SAS Institute), generating separate weights for each analyzed year.^19^ After applying the weights, beneficiaries in comparison states were similar on average to those in model states on the baseline demographic and health characteristics we examined (Table 1).

**Table 1.** Beneficiary Demographic and Outcome Summary Statistics at Baseline (Weighted)^a^

Characteristic	Mean		Difference (95% CI)
	Model	Comparison	
Age, y	71.5	71.5	−0.0 (−0.05 to 0.05)
Female, %	50.8	50.2	0.6 (0.44 to 0.76)
Race and ethnicity, %			
Black	30.4	29.8	0.6 (0.46 to 0.74)
White	64.2	64.7	−0.5 (−0.65 to −0.35)
Other^b^	5.5	5.5	−0.0 (−0.07 to 0.07)
Rural, %	20.2	20.4	−0.2 (−0.33 to −0.07)
Dual eligibility for Medicare and Medicaid, %	33.9	36.8	−2.9 (−3.05 to −2.75)
HCC score	4.3	4.3	−0.0 (−0.01 to 0.01)
Chronic condition, %			
ESRD only	56.2	56.3	−0.1 (−0.25 to 0.05)
Pressure ulcers only	41.7	41.6	0.1 (−0.05 to 0.25)
ESRD and pressure ulcers	2.1	2.1	0.0 (−0.04 to 0.04)
No. of beneficiaries	603 818	1 129 439	NA

Abbreviations: ESRD, end-stage renal disease; HCC, hierarchical condition category; NA, not applicable.

^a^ This table presents weighted means of beneficiary characteristics for beneficiaries with ESRD, pressure ulcers, or both. Comparison group individuals are propensity-score weighted to resemble model state individuals on baseline demographic and health characteristics.

^b^ Includes American Indian/Alaska Native, Asian/Pacific Islander, Hispanic/Latino, unknown, and other categories.

We estimated the association of RSNAT-PA with each of the outcomes using 2 methods: 2-way fixed-effects regression and event study. Our approach compared changes in outcomes between baseline and follow-up in the model states to changes in outcomes over the same time frame in the comparison states. Implementation year was defined for comparison states by the cohorts of their matched model states. In the 2-way fixed-effects regressions, state and year fixed effects control for long-term trends and for unmeasured state-level characteristics that do not change over time and might be associated with our outcomes (see eAppendix in the Supplement for a discussion of the underlying assumptions). To account for differences in beneficiary demographic and health characteristics that remain after weighting, our models also controlled for age, age squared, sex, race, rural residence, dual eligibility for Medicare and Medicaid, use of home health services, HCC score, an indicator for residing in a county with an active moratorium on new Medicare RSNAT suppliers, and length of time since the start of an active moratorium. We included the HCC score to control for the overall health risk of each beneficiary. We used logistic regression for binary outcomes and ordinary least squares for continuous and count outcomes.

We adjusted estimated SEs to account for association among multiple observations on the same individual. Analytic outcomes and subgroups were prespecified in our evaluation design report prior to beginning the study. All statistical hypothesis tests were 2-sided; a *P* value of less than .05 was considered statistically significant. Analyses were run in Stata, version 15 (StataCorp LLC).^20^

Following recent literature identifying the potential for bias in 2-way fixed-effects regression models,^21^ we conducted an event study analysis (estimating annual effects relative to a reference period), to better control for preexisting trends in outcomes and gauge the sensitivity of our results to heterogeneous treatment effects across states and years. We defined the reference period as the year immediately prior to the implementation of RSNAT-PA. All control variables and SE calculations were the same as in the 2-way fixed-effects analysis.

## Results

### Description of Study Population

Approximately 1.7 million Medicare beneficiaries were observed in the study (mean [SD] age, 71 [15] years; 50% female beneficiaries; 30% Black beneficiaries, 64% White beneficiaries; 20% rural residence; 35% dually eligible for Medicare and Medicaid; 58% with ESRD; and 44% with severe [stage 3 or 4] pressure ulcers). Our final study population consisted of 603 818 beneficiaries residing exclusively in model states and 1 129 439 beneficiaries who resided exclusively in comparison states. The number of years that each beneficiary was part of our sample ranged from 1 to 8, with a mean duration of 2.2 years. Approximately 1.7% of observations with missing beneficiary zip code and/or race and ethnicity data were excluded, leaving 3 583 232 beneficiary-years in the analysis sample. Missing rates were similar for the model and comparison state samples, 1.6% and 1.7%, respectively. The similarities in missing rates and the balance reported in Table 1 both suggest the data were missing at random. In the analytic sample of beneficiary-years, 69% represented beneficiaries with only ESRD, 28% represented beneficiaries with only pressure ulcers, and 3% represented beneficiaries with both conditions. In the baseline period, 7.1% of beneficiaries in the model states used RSNAT; in the comparison states, it was 4.9%. The difference reflects the selection of exceptionally high-use states into the model.

Across all years, beneficiaries in the model and comparison states were the same age (71.5 vs 71.5 years) and had the same prevalence of ESRD (56.2% vs 56.3%), pressure ulcers (41.7% vs 41.6%), and both ESRD and pressure ulcers (2.1% vs 2.1%) (Table 1). Model beneficiaries were slightly more likely to be Black individuals (30.4% vs 29.8%) and slightly less likely to be White individuals (64.2% vs 64.7%) . There was a slight difference in living in a rural area (20.2% vs 20.4%), and model beneficiaries were less likely to be dually eligible for Medicare and Medicaid (33.9% vs 36.8%). There was no difference on HCC scores (4.3 vs 4.3). See eTable 2 in the Supplement for weighted balance within each year observed.

**Figure 1.**
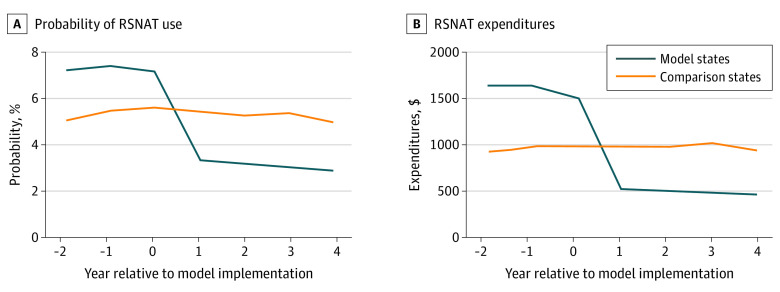
Probability of RSNAT Use and RSNAT Expenditures per Beneficiary per Year A, Probability of repetitive, scheduled, nonemergent ambulance transport (RSNAT) use. B, RSNAT expenditures.

Visual inspection of trends suggests that RSNAT-PA was associated with a sizable reduction in RSNAT use and expenditures. Figure 1 presents the unadjusted trends in use and expenditures for model vs comparison states in the 3 years before and 4 years after model implementation, which shows RSNAT states had a 58% decrease in unadjusted use and a 67% decrease in unadjusted expenditures once the model went into effect and essentially no change over this time-period for comparison states.

Adjusting for beneficiary and state characteristics in a 2-way fixed-effects regression framework, we found similarly sizeable decreases (Table 2). The RSNAT-PA Model was associated with a reduction in the probability of RSNAT use by 4.1 percentage points (95% CI, −4.26 to −3.94; *P* < .001) or 61% of baseline; the average number of RSNAT trips per year by 6.8 (95% CI, −7.05 to −6.55; *P* < .001) or 78%; RSNAT expenditures by $1136 per beneficiary per year (95% CI, −$1179 to −$1093; *P* < .001) or 77%; and total expenditures by $1530 per beneficiary per year (95% CI, −$1775 to −$1285; *P* < .001) or 2.4%. The effect sizes were largest for beneficiaries with ESRD only and smallest for beneficiaries with pressure ulcers only (subgroup results not shown).

**Table 2.** Association of RSNAT-PA With Use, Expenditures, and Quality of Care per Beneficiary per Year^a^

Variable	Average marginal effect size (95% CI) (n = 3 583 232 beneficiary-years)	*P* value	Baseline mean	Average marginal effect size as percentage of baseline mean
**Use and expenditures**				
Probability of RSNAT use (percentage points)	−4.1 (−4.26 to −3.94)	<.001	6.8	−61.0
No. of RSNAT trips	−6.8 (−7.05 to −6.55)	<.001	8.7	−78.4
RSNAT expenditures, $	−1136 (−1179 to −1093)	<.001	1477	−76.9
Total expenditures, $	−1530 (−1775 to −1285)	<.001	63 550	−2.4
**Quality of care per beneficiary per year**				
Probability of emergency department use	−0.99 (−1.17 to −0.81)	<.001	71.06	−1.39
Probability of emergency ambulance use	0.07 (−0.15 to 0.29)	.50	44.01	0.16
Probability of unplanned hospital admission	−1.53 (−1.71 to −1.35)	<.001	59.56	−2.57
No. beneficiary-years among beneficiaries with ESRD	2 525 702			
Probability of scheduled dialysis	−0.74 (−1.01 to −0.47)	<.001	62.46	−1.19
Probability of emergency dialysis	1.44 (1.28 to 1.60)	<.001	7.72	18.65
Probability of hospitalization due to ESRD complications	−0.94 (−1.08 to −0.80)	<.001	6.47	−14.47
Probability of unplanned hospital admission	−1.75 (−1.97 to −1.53)	<.001	51.09	−3.43

Abbreviations: ESRD, end-stage renal disease; PA, prior authorization; RSNAT, repetitive, scheduled, nonemergent ambulance transport.

^a^ The table presents average marginal effects from weighted logistic or ordinary least squares regression analyses representing service dates from 2012 to 2019. Coefficients from logistic regressions have been transformed into average marginal effects, which evaluate the effect of RSNAT-PA at the mean of all other control variables.

**Figure 2.**
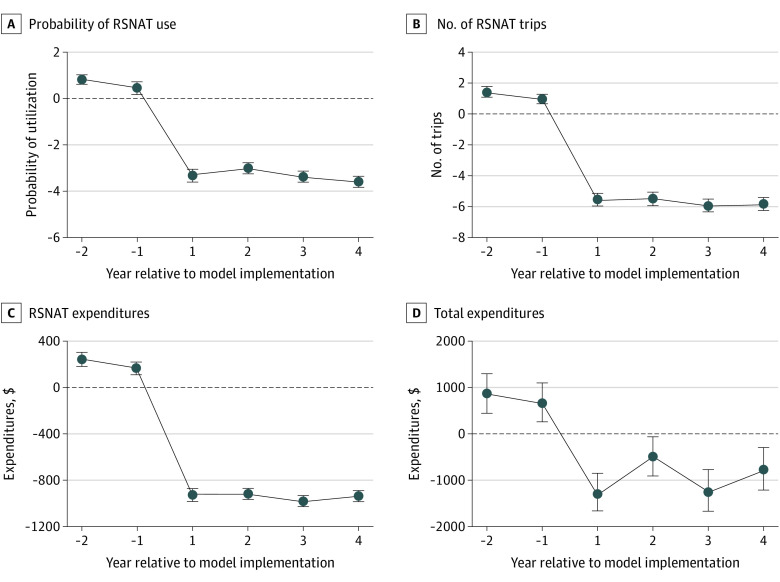
Event Study Estimates for RSNAT Use and Expenditures Event study estimates for probability of repetitive, scheduled, nonemergent ambulance transport (RSNAT) use (A), number of RSNAT trips (B), RSNAT expenditures (C), and total expenditures (D). The error bars represent 95% CIs.

The event study estimates were consistent with the results from the 2-way fixed-effects analysis; there were statistically significant reductions in all 4 outcome measures after the implementation of RSNAT-PA (Figure 2). However, in the 2 years prior to the reference period, all 4 measures were significantly higher, which suggests a possible anticipation effect, with RSNAT use and expenditures decreasing in the model states in the year immediately prior to implementation. Because the event study uses that year as the reference period, this analysis may underestimate the associations between RSNAT-PA and these outcomes, whereas the 2-way fixed-effects analysis considers the effect of RSNAT-PA using the full preintervention time period. For example, the 2-way fixed-effects analysis estimated a reduction of 4.1 percentage points in the probability of a RSNAT trip per year, whereas in the event study this estimated reduction ranged from 3.0 (95% CI, 2.84-3.16) to 3.6 (95% CI, 3.45-3.75) percentage points over the follow-up period. Despite small differences in the magnitude of the estimates, both analyses suggest the same conclusions about the RSNAT-PA Model.

**Figure 3.**
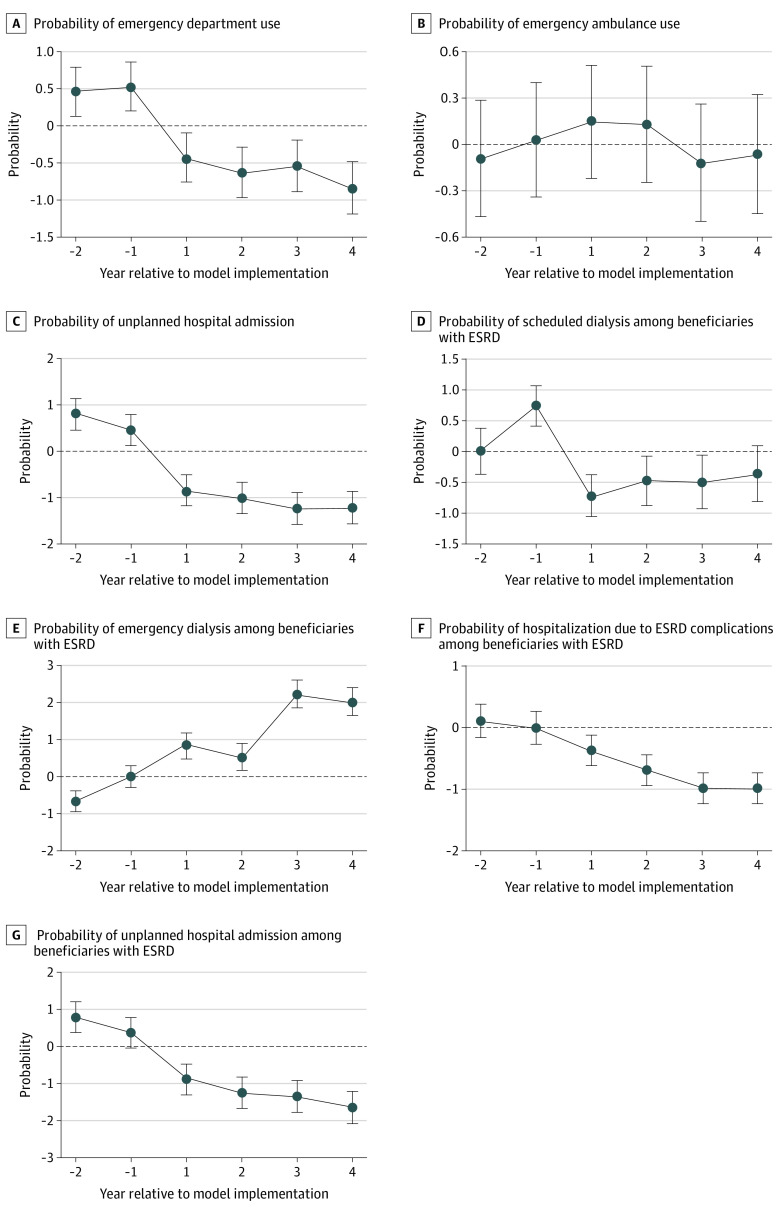
Event Study Estimates for RSNAT Quality of Care Outcomes Event study estimates for probability of emergency department use (A), probability of emergency ambulance use (B), probability of unplanned hospital admission (C), probability of scheduled dialysis among beneficiaries with ESRD (D), probability of emergency dialysis among beneficiaries with ESRD (E), probability of hospitalization due to ESRD complications (F), probability of unplanned hospital admission among beneficiaries with ESRD (G), and probability of unplanned hospital admission among beneficiaries with ESRD. ESRD indicates end-stage renal disease; RSNAT, repetitive, scheduled, nonemergent ambulance transport. The error bars represent 95% CIs.

In the full study population and using the 2-way fixed-effects framework, we found no evidence suggesting that RSNAT-PA reduced quality of or access to care (Table 2). Beneficiaries were not more likely to use emergency transportation services (0.07 percentage points, 95% CI, −0.15 to 0.29; *P* = .50), and our estimates were consistent with a small decrease in the probability of emergency department use (−0.99 percentage points, 95% CI, −1.17 to −0.81; *P* < .001; −1.4%) and unplanned hospital admissions (−1.53 percentage points, 95% CI, −1.71 to −1.35; *P* < .001). The event study also showed no significant change in emergency transportation use ranging from −0.07 (95% CI, −0.46 to 0.32) to 0.14 (95% CI, −0.23 to 0.51) percentage points and small decreases in emergency department use and unplanned hospital admissions (Figure 3).

Among beneficiaries with ESRD, the 2-way fixed-effects estimates suggested that there was a small decrease in scheduled dialysis use (−0.74 percentage points, 95% CI, −1.01 to −0.47; *P* < .001), and a small increase in emergency dialysis use (1.44 percentage points, 95% CI, 1.28 to 1.60; *P* < .001). Hospitalizations for complications of untreated ESRD decreased slightly by 0.94 percentage points (95% CI, −1.08 to −0.80; *P* < .001), and unplanned hospitalizations also decreased slightly (−1.75, 95% CI, −1.97 to −1.53; *P* < .001).

The event study also showed a small decrease in scheduled dialysis use, with no significant difference by year 4 (−0.35, 95% CI, −0.79 to 0.09), as well as an increase in emergency dialysis use after year 2 (Figure 3). Event study estimates for hospitalizations for complications of untreated ESRD and unplanned hospitalizations were consistent with the 2-way fixed-effects estimates.

## Discussion

In this difference-in-differences analysis, we examined the impact of prior authorization for a service vulnerable to improper use. We assessed the association with total cost of care, service use and cost, and a series of well-established health care outcomes. We found evidence suggesting that RSNAT-PA was associated with reduced total cost of care and substantially reduced RSNAT use and cost, without appearing to be associated with changes in most of the quality or access indicators we examined. We did find evidence of a decrease in scheduled dialysis, amounting to 1% of baseline, suggesting the possibility of limited delay of care and an increase in unscheduled dialysis visits, approximately 19% higher than baseline. However, we did not find evidence of adverse effects on downstream patient outcome measures, such as hospitalizations. Nevertheless, we recognize that emergency dialysis use can be stressful for the patient and could lead to more serious outcomes if it were to occur repeatedly, such that this result may warrant ongoing monitoring. This finding also suggests that there may be opportunities to improve the prior authorization process to avoid this result in the future. One such improvement would be to identify the reasons for emergency dialysis use among these beneficiaries and determine if beneficiaries with the greatest potential transportation challenges (such as those with limited or no access to public transportation services or those with the greatest distances to travel for medical care) or those who were more fragile but did not meet Medicare’s medical necessity criteria were more likely to require this service. If warranted, adjustments to the prior authorization requirements for these beneficiaries may reduce the potential for negative effects on access to care and patient health.

The event study estimates similarly suggested large decreases in use and expenditures without adverse outcomes. A small decrease in use prior to the start of the pilot program indicates an anticipatory effect, resulting in attenuated overall outcome estimates compared with the 2-way fixed-effects analysis. To the extent this effect was real, by capturing the full preimplementation time frame as the reference, the 2-way fixed-effects analysis provides a more comprehensive assessment of the association of RSNAT-PA with these outcomes, as by design, any anticipatory effect was part of the model.

The savings from RSNAT-PA, estimated at approximately $1 billion in total Medicare costs for the period of 2015 to 2019, are far larger than the estimated administrative costs to CMS of less than $40 million per year.^22^ These findings were robust to several sensitivity analyses and served as the basis for CMS’s decision to expand the program nationally.

Our results suggest that prior authorization could be successfully expanded to other Medicare services, specifically services that are vulnerable to improper use when those services are not medically necessary. Prior authorization enables payers to balance the need to closely monitor for unnecessary use and potential fraud with the goal to ensure proper care for patients. When targeted, prior authorization can be an effective and appropriate cost-containment strategy.

Future studies of the outcomes of prior authorization might be of greatest use to policy makers if they explicitly include both total cost and patient health outcome measures and use the most rigorous research designs possible. These studies should include not only cost measures for the service subject to prior authorization, but also the total cost of care to confirm that no countervailing increase in another service occurs. An increase in total cost of care might indicate an adverse outcome on patient health, leading to increases in other medical services. In addition, several explicit patient outcomes and quality of care measures should be included in these studies to provide a detailed view of potential negative impacts on beneficiaries. Most studies to date have focused on outcomes on use and costs of the targeted service, not total cost of care or patient outcomes or quality of care measures.

### Limitations

As noted previously, the states selected for RSNAT-PA had the highest rates of use for RSNAT in the nation. This selection drives 2 important limitations. First, our evaluation relied on a quasi-experimental design in which constructing an appropriate comparison group was critical. Our design used state-level matching, beneficiary-level weighting, and regression controls to reduce differences between the intervention and comparison groups. Second, though the evidence suggests that prior authorization was associated with reduced overuse with little to no association with quality and access to care, we expect the national rollout of RSNAT-PA to have a smaller impact on use and costs because of lower baseline rates of improper use in the rest of the nation.

## Conclusions

In this difference-in-differences analysis of Medicare beneficiaries, we evaluated the association of prior authorization with unnecessary use and cost for a Medicare service vulnerable to improper use. Our findings suggest that there was a dramatic reduction in use of and payments for RSNAT services, with no evidence of an increase in emergency ambulance use, emergency department visits, unplanned hospital admission, or total cost of care, suggesting that much of the high RSNAT use at baseline was overuse. This evidence suggests that prior authorization may be an effective strategy in reducing improper use of health care services when targeted appropriately.

## Notes

Supplement. **eAppendix**. Difference-in-differences inferential assumptions **eTable 1**. State-level matching results **eTable 2**. Standardized differences between model state and comparison beneficiaries before and after inverse propensity score weighting
