# Supplementary material for: Evaluation of Prior Authorization in Medicare Nonemergent Ambulance Transport
Source: JAMA Health Forum. 2022 Jul 15;3(7):e222093. doi: 10.1001/jamahealthforum.2022.2093 (PMC9287758; doi:10.1001/jamahealthforum.2022.2093)
Supplement: Supplement. — eAppendix. Difference-in-differences inferential assumptions eTable 1. State-level matching results eTable 2. Standardized differences between model state and comparison beneficiaries before and after inverse propensity score weighting [file jamahealthforum-e222093-s001.pdf]

Supplemental Online Content

Contreary K, Asher A, Coopersmith J. Evaluation of prior authorization in Medicare nonemergent ambulance transport. *JAMA Health Forum.* 2022;3(7):e222093. doi:10.1001/jamahealthforum.2022.2093

eAppendix: Difference-in-differences inferential assumptions

eTable 1. State-level matching results

eTable 2. Standardized differences between model state and comparison beneficiaries before and after inverse propensity score weighting

This supplemental material has been provided by the authors to provide readers additional information about their work.

© 2022 Contreary K et al. *JAMA Health Forum.*

eAppendix: Difference-in-differences inferential assumptions

Under certain conditions, difference-in-differences estimate can be interpreted as the causal impacts of RSNAT-PA because our statistical models control for unmeasured characteristics that do not change over time. The key assumptions are: (1) absent the model, the difference between model and comparison groups would remain constant over time (i.e., parallel trends); (2) the types of beneficiaries in the study population did not change over the study period; and (3) there were no spillover effects. The approach also requires that there were no other changes in the policy environment during the study period that could affect our outcomes of interest differently in the model and comparison states. We found support for the assumption of parallel trends in outcomes—plots of the yearly trends in key outcomes in model and comparison states revealed very similar patterns during the three- or four-year pre-model period (see, for example, Figure 1). Spillovers were unlikely because ambulance services tend to be provided at a very local level and few comparison states bordered model states. (Any cross-border activity would tend to be in the direction of evading prior authorization, biasing our estimates towards zero.) We also examined the Medicare policy environment over the study period and found no specific policies that were likely to affect our key outcomes of RSNAT use and expenditures. However, some policy changes might have modestly affected some of the quality and access outcomes related to hospitalization we examined.[19]

The estimated impacts of RSNAT-PA on utilization and cost are quite large, making it unlikely any unmeasured confounding factor could explain this effect. Thus, the combination of the matching and comparison group construction, the weights, the analysis approach, and the estimated impacts provide good reason to doubt the possibility of any unmeasured confounding factor explaining the effects presented here.

© 2022 Contreary K et al. *JAMA Health Forum.*

eTable 1. State-level matching results

		All non-RSNAT-PA states		Matched comparison states	
Measure	RSNAT-PA states	Mean	Standardized difference	Mean	Standardized difference
RSNAT utilization	1,716	508	-0.87	954	-0.55
Change in RSNAT utilization 2012-2013	+151%	+57%	-0.22	+187%	0.08
Number of ambulance suppliers per 100,000 Medicare beneficiaries	9.4	4.3	-0.79	6.9	-0.38
Percentage of Medicare beneficiaries with ESRD	1.5	1.2	-0.64	1.3	-0.32
Average age of Medicare beneficiaries	71.0	70.9	-0.02	70.6	-0.44
Percentage of Medicare beneficiaries living in rural areas	24.7	38.1	0.73	33.9	0.50
Percentage of Medicare beneficiaries living in nursing homes in 2012	2.5	2.8	0.89	2.9	1.4

Note: Unless otherwise specified, all measures use 2013 as the pre-intervention baseline. Table values represent state-level means and do not account for the population size of the states.

© 2022 Contreary K et al. *JAMA Health Forum.*

eTable 2. Standardized differences between model state and comparison beneficiaries before and after inverse propensity score weighting

	Percent living in rural areas		Percent female		Percent white		Percent black		Percent with ESRD		Percent with cancer		Percent with skin ulcers	
	Before IPT	After IPT	Before IPT	After IPT	Before IPT	After IPT	Before IPT	After IPT	Before IPT	After IPT	Before IPT	After IPT	Before IPT	After IPT
2012	0.04	0.00	0.00	0.00	-0.08	0.00	-0.13	0.00	0.06	0.00	-0.06	0.00	-0.05	0.00
2013	0.04	0.00	0.01	0.00	-0.09	0.00	-0.13	0.00	0.06	0.00	-0.06	0.00	-0.06	0.00
2014	0.04	0.00	0.00	0.00	-0.09	0.00	-0.12	0.00	0.06	0.00	-0.06	0.00	-0.06	0.00
2015	0.04	0.00	0.00	0.00	-0.08	0.00	-0.12	0.00	0.09	0.00	-0.06	0.00	-0.09	0.00
2016	0.05	0.00	-0.01	0.00	-0.08	0.00	-0.12	0.00	0.08	0.00	-0.06	0.00	-0.08	0.00
2017	0.05	0.00	-0.01	0.00	-0.08	0.00	-0.12	0.00	0.08	0.00	-0.06	0.00	-0.08	0.00
2018	0.05	0.00	-0.01	0.00	-0.08	0.00	-0.12	0.00	0.09	0.00	-0.06	0.00	-0.09	0.00
2019	0.05	0.00	-0.01	0.00	-0.08	0.00	-0.12	0.00	0.08	0.00	-0.06	0.00	-0.08	0.00

© 2022 Contreary K et al. *JAMA Health Forum.*
